# “Discrimination is always intersectional” – understanding structural racism and teaching intersectionality in medical education in Germany

**DOI:** 10.1186/s12909-023-04386-y

**Published:** 2023-06-02

**Authors:** Merle Weßel, Simon Matteo Gerhards

**Affiliations:** Ethics in Medicine, Carl von Ossietzky University of Oldenburg, Ammerländer Heerstraße 114-118, 26111 Oldenburg, Germany

**Keywords:** Intersectionality, Structural racism, Medicine, Health care, Qualitative research, Germany, Medical students

## Abstract

**Background:**

Racism in medicine represents a global problem. It takes place on the individual, institutional and structural level. Especially structural racism can have serious effects on the health of individual people. Furthermore, racist discrimination is not always based on race solemnly but frequently intersects with other social categories such as gender, class or religion. To describe this multidimensional form of discrimination the term intersectionality has been coined. However, the understanding of structural intersectional racism in medicine is still fragmented, especially in the German context. Yet, medical students need to be trained in understanding structural and intersectional racism to see the impact of racist structures on the patient’s health.

**Method:**

We conducted a qualitative study to explore the knowledge, awareness and perception of racism in medicine and health care of medical students in Germany. Our research questions are how do medical student understand structural racism and its effects on health in Germany? Do students see interrelations with other forms of discrimination and in this context to what extent are they familiar with the concept of intersectionality? Which categories intersect from their point of views with race in context of medicine and health care? We conducted focus groups with medical students (n = 32) in Germany.

**Results:**

Our results demonstrate that students have a wide spectrum of knowledge, awareness and perceptions about racism from being rather elaborate to very little knowledge about it. The students have particular problems to understand and situate structural racism in Germany. Some raised doubts about the relevance. Yet, other students are aware of the concept of intersectionality and are convinced that racism must be looked at from an intersectional perspective.

**Conclusions:**

The diverse knowledge, awareness and perceptions of medical students about structural racism and intersectionality hints to a lack of systematic education of medical students about these issues in Germany. Yet, in context of diversifying societies an understanding about racism and its impact on health is imminent for future medical doctors to provide good care for their patients. Therefore, this knowledge gap must be systematically filled by the medical education.

## Background

Discrimination constitutes a form of unequal treatment based on a (perceived) belonging to a group without a legal or ethical reasoning for it. For example, when access to an institution is denied because of the skin color of a person or unequal pay on the labour market due to the gender of a person [1]. Racism is a central form of discrimination and inequality in medicine and health care. It influences the access to and quality of health care but also health itself. For understanding how racism affects health not only on the interindividual level but its foundation on unequal structures and multidimensional factors, recent research often refers to the concepts of structural racism [2–10] and intersectionality [11–15]. In the US-context first attempts have been made to integrate structural and intersectional approaches to racism in anti-racist policies [16] and medical education [17, 18]. Also, in the *EU anti-racism action plan 2020–2025* the European Commission acknowledges the role of structural racism, highlights the need for an intersectional approach and calls for research to be “undertaken on the socio-economic determinants of health from the racial perspective” [19]. This suggests that the problem of structural racism on health is recognized and this paper contributes to this debate by discussing what role medical education should play by tackling this issue.

Meanwhile, only scarce research exists about structural and intersectional racism in medicine and health care in Germany [20]. Moreover, as the concepts of intersectionality and structural racism originate in the United States, the transfer and implementation of these concepts in the German context is due to societal, national and historical differences not as such possible and they need a reconceptualization. To understand structural and intersectional racism in medicine and health care in Germany we conducted focus groups with medical students (n = 32) to explore their knowledge, awareness, and perceptions of racism. Medical students are a particular relevant group since they are the next generation doctors but they also have a unique view on the medical education as well as medical practice due to their all-encompassing experiences in both areas. The discussion groups covered interindividual, institutional and structural racism as topics. This paper focuses on their perspectives on structural racism as well as the interaction of racism with other forms of discrimination in context of intersectionality. Our research questions are how do medical students understand structural racism and its effects on health in Germany? Do students see interrelations with other forms of discrimination and in this context to what extent are the medical students familiar with the concept of intersectionality? Which categories intersect from their point of views with race in context of medicine and health care?

Racism holds specific features in different social and historical contexts, e.g. in Europe or USA [21, 22]. Nevertheless, bringing together her insights from context specific studies of racism in the Netherlands and USA, Philomena Essed defines racism as “an ideology, structure and process by which certain group belongings are regarded as inherently different and inferior ‘races’ or ethnic groups on the basis of actual or ascribed biological or cultural characteristics. Subsequently, these differences serve to explain the exclusion of members of these groups from access to material and non-material resources” [23, 24]. The forming of groups and the hierarchization in “us” and “them” based on ascribed and essentialized biological or cultural characteristics is also called *racialization* [25–27]. This term underlines the fact that “races” do not exist biologically but are the product of racism as an ideology [28, 29].

While racism always has to do with power relations among racialized groups [30], there might be contextual differences regarding which groups are oppressed by racism [24]. While in the US-context racism concerns in the common sense primarily racial groups as they are identified by the US-census such as Black, Asian and White, in the German context a “racism without races” is observed by scholars [31]. This racism – also called “cultural racism” – refers additionally to biological race-notions to racialization based on among others perceptions of migration, nationality, culture, or religion [32]. In Germany, discourses on race and racism have long been considered as something of the past mainly related to the time of National Socialism and notions of racial theory of the 19th and early 20th century. After 1945 race and racism has been discussed little and insufficiently conceptualized. Just recently new publications have taken up the issue of the lack of conceptualization of race and racism in Germany and demonstrate that racism indeed exists in Germany but was long covered by an anxiety about the historical past and masked with xenophobic concepts based on nationality and religion [33, 34].

Also, the research on racism in the context of health care often lacks thorough definitions and conceptualizations [30, 35]. In the US-context models were developed to explain the effects of racism on health such as Nancy Kriegers’ ecosocial theory [36] or the idea of a “weathering” by the effects of racist discrimination [37]. Another concept of growing interest is “structural racism” [10, 38]. In social sciences concepts of structural racism have been developed already in the 1990s [3, 39]. Previous understandings of racism have been criticized for focussing too much on interpersonal and individual aspects such as ideological beliefs or prejudices [39, 40]. Instead, structural approaches to racism aim for a comprehensive understanding of the underlying societal mechanisms that shape materialistic inequalities between racialized groups [39–41].

In health research a structural approach comes with the aim to identify reasons of unjust health inequities between racialized groups in structural conditions and to derive recommendations for countermeasures [8, 36]. Still, there is an ambiguity and a wide heterogeneity of definitions and measurements of structural racism that shape the public health and medical research discourse [3, 10]. Often, the terms of structural and institutional racism are used interchangeably [e.g. 42] but in recent public health research these terms are used to name different levels. The term “institutional racism” is there and in this text reserved for “racism within a particular type of institution” [10] such as educational or healthcare institutions. While acknowledging the preceding defining works on the terms of structural and institutional racism, Dean and Thorpe propose to define structural racism as the “totality of ways in which multiple systems and institutions interact to assert racist policies, practices, and beliefs about people in a racialized group” [7, 10]. In the Anglo-American context research on structural racism can lean on established social categories such as race and ethnicity, and identified domains of institutional racism (e.g., housing, education, employment, earnings, media, health care, criminal justice [43]) that together interact as structural racism. In contrary, in the German context, research on structural racism and health inequity lacks these preconditions and therefore comes along with a variety of open questions [44, 45].

Nevertheless, in order to understand health inequities, not only different pathways of materialized injustice between racialized groups have to be taken into account but also the interaction of multiple different types of injustices [36]. This phenomenon is represented by the concept of intersectionality [46]. A sole focus on the category of race in the context of structural racism has been criticized as short sighted since it hides the heterogeneity of the experiences of racism. The Black feminist [47, 48] theory of intersectionality has pointed out that the particular discrimination Black women experience remains unseen in ordinary discourses about racism [46, 49]. In the late 1980s, the term intersectionality was then coined by the legal scholar Kimberlé Crenshaw [46]. Intersectionality describes structural discriminations that are based on more than one social category and cannot be described in concepts of racism, sexism or classism. For example, a Black woman does not experience discrimination due to her gender *or* her race but due to her gender *and* her race. Both categories intersect equally and create the inequalities she experiences. However, neither racism nor sexism can describe this multidimensional discrimination, so that Crenshaw created the term intersectionality to denominate co-constitutive, non-hierarchical, multidimensional discrimination funded in power structures. Since then, intersectionality has been developed into a traveling concept that does not only focus on the categories of gender and race but on various intersections of gender, class, sexuality, disability, age, education to name only few to describe multidimensional, non-hierarchical structural discrimination [50].

Especially when investigating health disparities in context of public health intersectionality has long been used to describe multidimensional structural discrimination with a focus on race [14, 51–53]. Nevertheless, also other areas of healthcare research, such as medical ethics [e.g. 15, 54, 55] or medical education [56–59], have discovered intersectionality as useful concept. However, in the German context a comprehensive understanding of intersectionality in medicine and health care is still a research gap. First studies pick up on the subject [e.g. 60] but a coherent conceptualization of intersectionality for the German context in medicine and health care is still lacking. Yet, our study provides a first exploration of structural and intersectional racism in the context of medical education. Our results give insights about medical student’s perceptions of these concepts. Our findings are discussed with regard to the integration of the concepts of structural racism and intersectionality in medical education curricula in Germany.

## Method

Due to the explorative nature of the subject, we opted for a qualitative approach. Between July and September 2021, we conducted six focus groups with medical students (n = 32) from all over Germany. The sample size represents data saturation. Due to the explorative character the aim was to keep the sample size to a manageable number to ensure more of an in-depth analysis. Furthermore, due to the online setting, we decided to keep the number of participants per group low to facilitate a good discussion atmosphere. The inclusion criteria were the enrolment in medical studies at a German university, the age of majority and sufficient German language skills to follow and contribute to the discussion. The recruitment of the participants took place via local student bodies, offices of student affairs, lecturers, and informal student groups, as well as via social media. Furthermore, snowball sampling was pursued. A pre-questionnaire was used to collect socio-demographic information (age, gender, term, discrimination experience, political activities). Participants provided written informed consent in advance. IRB approval was obtained from the ethics committee of the Medical Faculty of the Carl von Ossietzky University of Oldenburg (No. 2021-080).

Of the 32 participants eight identified as male and 23 as female, one person as diverse. The age of the participants ranged from 18 to 31 years old, and they studied from year one to year six. Six of the participants reported that they experienced racism themselves. The declaration of the experience of racism was a self-assessment of the participants. It was not asked what type of racism they had experienced rather if they experienced any kind of discrimination and six participants stated that they experienced discrimination due to their race, ethnicity, or background which we then defined as racism. Twelve participants noted down that they were politically active.

In the development of our study, we thought about the safety of those who experience racism. Since the aim was to study the dynamics of group discussions about racism as they might come up in any context of medical education, the groups were not divided into students with and without personal experiences of racism. The group discussions came along with the danger of re-experiencing racism, especially as medical students in Germany are not used to discuss racism openly in the context of their studies. Taken precautions included the obtained written consent of all participants into participating in discussions about racism and informing the participants that re-experiencing racism and microaggressions in the discussions cannot be ruled out entirely. Moreover, the participants were asked to interact respectfully. The moderation team was prepared to intervene and were during the online discussions continuously available for the participants’ feedback via direct messages. A list of contact persons and anti-discrimination organisations was provided to all participants.

Moreover, it is important to consider the possible influences of our positionalities and perspectives on the whole research process [61]. We, the two authors, self-identify as *white* and are also perceived by others as *white*. We therefore are structurally privileged by racism. This positionality might have influenced the way participants talked about racism in group discussions which we moderated and makes us especially susceptible for blind spots in the analysis of our data e.g., regarding specific experiences of non-*white* racialized people or our own racist views and biases. We met this challenge by intensive exchange with colleagues in different contexts and from different working groups who embody more diverse perspectives on the topic. We chose the perspectives of medical students on racism as research focus because our involvement in medical ethics teaching made us think about approaches to critically address the topic of racism in medical education.

The focus group discussions were conducted online via the video conference tool Webex. They lasted between 90 min and two hours and were guided by two moderators. A semi-structured guideline was used in the groups. The guideline included topics of own experiences or witnessing of racism, as well as questions regarding the awareness and understanding of interindividual, institutional and structural racism. Furthermore, the participants were asked to make suggestions how medical education could be improved in context of anti-discrimination teaching. We purposely did not use the term intersectionality in the guideline but asked about how racism interacts with other forms of discrimination and what kind of people experience discrimination from the point of view of the participants. The aim was to find out if the participants are familiar with the concept of intersectionality and can name it without us explicitly talking about it. The discussions were audio recorded, the recordings transcribed verbatim and anonymized. Then we conducted a thematic qualitative content analysis; in a second step we conducted an intersectional analysis [62, 63].

Since we had a large amount of data demonstrating various aspects in context of racism, we firstly used the thematic qualitative content analysis to organise the data (see Table 1 for illustration of the coding process). This thematic qualitative analysis was carried out by two researchers, one postdoctoral researcher in the field of medical ethics and one medical PhD student. Following the guidelines for a thematic qualitative analysis we firstly went jointly through the data to get a first impression and mark relevant text passages. Then we developed guided by our discussion guidelines deductively the codes for a first coding. For the first round of the coding the data was divided between the two researchers but they worked closely together and discussed their progress in regular meetings to ensure common results. During the first round of coding the researchers already created memos to prepare the creation of inductive codes. After the first coding was finalized the researchers met and decided about further inductive codes together. This was followed by a second round of coding.

After the second round of coding was finalzsed, the intersectional coding process was continued by the postdoctoral researcher, since she is an expert on intersectionality. One of the inductive codes created in the previous round was “intersectionality”, under which we coded when participants explicitly named intersectionality or discussed the interrelation of forms of discrimination. Also, she chose to include the code of “structural racism” into the intersectional coding, because structural racism and intersectionality are closely entangled as concepts since intersectionality describes the impact of power structures on people experiencing multicategorical discrimination. The code “intersectionality” was first coded to extract the understandings and evaluations of the concept of intersectionality of the participants. Then in the second round the text passages with the deductive codes “intersectionality”, “understanding of racism”, “definition of racism” and “structural racism” were coded inductively to extract the social categories which from the point of view of the participants intersect with racism. By using an inductive method, the demands of a bottom-up approach of an intersectional analysis were met [64].

**Table 1:** Illustration of the coding process

Codes	Themes	Subthemes	Results
Intersectionality Structural racism Understanding of racism (with subcode affected groups) Definition of racism	Structural racism	Results of structural racism	- understanding of structural racism - evaluation of relevance in Germany - problematization of the term “race” in Germany - influence of related categories, such as migration or ethnicity
		Structural discrimination categories	
		Evaluation of structures	
	Intersectionality	Class	- knowledge and definition of the concept of intersectionality - naming of categories that intersect with race and create multidimensional discrimination
		Religion	
		Sexuality	
		Education	
		Geography	
		Gender	
		Migration	
		Skin color	

## Results

The knowledge, awareness, and perceptions of structural racism and intersectionality of the participants range on a spectrum from rather elaborate understanding to very little knowledge about the relevance of structural racism in German medicine and health care. Some also stated doubts about the relevance of structural racism in Germany. The understanding of the entangled concept of intersectionality demonstrates a similar result. We structured our description of the results in the following way. We firstly show the understanding of structural racism of the participants. For this we used different charts as prompts that displayed health statistics including child death rate [65], death rates in context of heart diseases [7] and in context of COVID19 [66] differentiated by US-census race/ethnicity groups. The students were asked to discuss the statistics and if they could imagine similar phenomena in the German context. We used charts from the US-American context because we assumed that the participants would be more familiar with the concept of structural racism in the US-context and because we were interested in how they would then discuss comparable phenomena in the German context. Since intersectionality is a concept which describes multidimensional structural racism, we then show the spectrum of understanding of the participants and what social categories they named as relevant in context of intersectional racism in medicine and health care.

### Knowledge, awareness and perceptions of structural racism

The responses of the students showed a spectrum of knowledge and attitudes about structural racism which ranged from a rather recessed understanding of structural racism and its effects on health to students who had problems to contextualize what they observe in the statistics. Others rejected the idea of structural racism altogether and searched for alternative explanations, for example cultural aspects. The transfer of the concept of structural racism from the United States to Germany constituted a particular challenge.

The students with a more elaborate understanding of structural racism were able to contextualize what they observed in the charts. One participant gave a rather extensive analysis of the statistics they saw: “For me that is a quite impressive picture, which is indeed representative for the global context, that certain risk marker or risk factors are connected with being assigned to a group partly coming from colonial times” (1, 71). Another student analyzed: “And sometimes I do not know (…) if structural racism is obtained to preserve power hierarchies, which are comfortable for so many people” (4, 37). This person showed no doubts about the existence of structural racism and suggests it is maintained to preserve power structures and the own advantageous status. Another one suggested the importance of research on structural racism: “Also, that just by this type of research one found out and could show that racism is a social construct with very clear consequences” (2, 124). Others were not familiar with the concept but showed interest in it. One participant stated that the idea of structural racism was new to them but they acknowledged it as an important new insight they were gaining from interpreting the statistics together with the other participants: “Okay, also I find that super interesting to hear that, because I did not have this thought but I rather interpretated it that in your racist systems these division are made, which I also find kind of important (…)” (2, 119). Though the person saw that certain constructions of groups can contribute to structural racism, they also understood it as an important tool to show the differences between groups and the effects of discrimination on health.

Others were more hesitant to acknowledge the role of racist structures on health and contribute different health status of ethnic groups to for example cultural aspects, like nutrition. One student speculated “that also always a certain ethnical segregation could play a role (…) generally is the Asian food culture known for less calories and higher nutrition, maybe that could play a role (…)” (3, 63). Another person agreed on that: “I think that Asian food often has less meat than that from Black or white Americans (…)” (3, 64). These participants had issues to understand the concept of structures, for example social structures, and they were not familiar how the concept is defined which made them look for other seemingly unproblematic forms of categorization like cultural aspects.

### Transfer of structural racism to the German context

Next the students were asked to consider similar phenomena in the German context. This transfer was especially difficult for all of them. One student answered bristly: “I do not understand why this shall be different than in the USA?” (1, 111), because they did not believe that societal, political and national contexts influence structural racism, but that race and racism was conceptualize globally in the same way. Others found it generally hard to accept that structural racism, especially in Germany, exists:

I find it hard to believe that they treated worse because of that [racism]. Also, I hope that treatment is not worse because of different skin colors. (…) instead it hopefully is just an expression of their socio-economic status. (…) also, that there are at least bigger reasons and not structural racism in medical care (1,75).

They wondered what categories beside race, which is not a term commonly used in the German context for historical reasons, could demonstrate the appearance of structural racism in Germany. Socio-economic status, migration background and education were three options they came up with when discussing structural racism in Germany. One participant reasoned:

To be honest, I find that question a bit difficult, since I believe in Germany definitely classism and racism play a role (…) sometimes the socio-economic status can be an advantage and in other cases the socio-economic status is not an advantage (…) when people are disadvantaged due to their migration background in the health system (4, 81).

This student related the socio-economic as well as a migration background to the experience of disadvantages in health care but is unsure how to conceptualize it in terms of racism or other forms of discrimination. Another one wondered if a lack of data was an issue in Germany to demonstrate the state of structural racism:

I think so too that there is no other data in Germany, maybe just adjusted to quantitative make-up of the population. (…). But I personally have the feeling that in Germany there is not so much talk about ethnic affiliation or race than in the USA, instead here it’s much more looked at the socio-economic status (…) (1, 111).

Also, the term migration background was used to describe racialization in Germany:

I would say, also in Germany its less the division between Black and white but rather people with and without migration background. And I think that especially the Global South plays in Germany a bigger role than people with a migration background from Eastern Europe (4, 87).

These participants argued that structural racism is indeed an important, though underresearched and overlooked problem in Germany but that the relevant term is not race but rather socio-economic status or migration background. This might make it harder to relate e.g., health differences in the first place with structural racism and masks it as another phenomenon than racism, for example poverty. They negotiated which terms and groups belongings should be used in context of racism in Germany.

### Intersectionality and racism

Though intersectionality was not a topic explicitly asked about in the focus groups, it was a topic that came up eventually in the groups addressed by the participants when asked to what extent racism interacts with other forms of discrimination. “Discrimination is always intersectional, also the more discrimination marker one has the more affected you are. (…) And I think that these intersectional components must always be considered” (1, 77) one participant for example claimed. The focus only on (structural) racism was not considered enough for them but they argued that racist discrimination must be looked at in connection with other social categories:

I think especially affected so generally are firstly groups that are discriminated based on various aspects. Maybe because they are female, transsexual, or maybe with a dark skin and also from a worse socio-economic background, also when this is all applies, I think, that it is especially extreme (3, 38).

Though the participants were familiar with the term, they had problems to conceptualize it:

But I have mentioned intersectionality before and I find it very hard to differentiate also how I can separate that I am a woman and I might be affected by discrimination, I cannot separate a white or Black person from their socio-economic background. That is a conglomeration and many aspects are connected with another (4, 79).

They were unsure of the correct use: “is that not a little bit like a little bit like this approach of intersectional discrimination. (…) But one can be discriminated once, when one is Black, one can be discriminated double also intersectionally, when you are Black and poor and maybe also homosexual.” (4, 56). This student had heard the term intersectionality but had trouble explaining the exact content of the definition. They were lacking the conceptual knowledge to define intersectionality as a concept and apply it to the analysis of health data for example or to the individual situation of a patient. However, none of the participants denied the existence of intersectional discrimination or the particularity of the effects of several discrimination categories on one person.

### Relevant social categories intersecting with racism

Yet, when discussing intersectionality, they found various social categories that intersected with race, for example class, gender, education, religion or migration. The participants drew up connections between the intersections of social categories, racism and the effects on health:

or what one perhaps can call institutional discrimination is the lack of effective preventive medical measures affects in particular people with worse socio-economic pre-conditions under which also many people with a migration background are (…) (3, 46).

Especially the migration background was in the German context strongly intersected with racism by the participants. They argued that racism against people with a migration background was often not made visible in Germany:

(…) there is very little data about people with a migration background, however you define this category. Until 2017 or so. And then also people with a migration background were included into studies, and I think, that is indeed the category which is used in Germany. Also, one says people with migration background and means people migrated in the first or second generation (…) (2, 148).

The perceived conceptual unclarity of migration led in the eyes of the student to the fact that also very little was known about the situation of migrants and how the category of migration intersected with racism.

The interlinking of religion and racism, especially anti-islamic racism, was discussed especially on the interindividual level. The students had witnessed that Muslim patients and colleagues were treated differently and experienced direct racism by medical staff or patients due to their religious symbols. One student remembered “situations in which people with a different skin color, or religious symbols of I might say here non-traditional religions have been described with terms like *Morbus Mediterraneus*.” (1, 24). Another participant explained: “and also I have witnessed anti-islamic racism. Because there were colleagues with a headscarf. (…) that was really drastic (the behaviour) from some patients” (1, 34).

A further category which was named by the participants and intersected with race is education. One participant equated the effects of a lower class background with low education: “That is certainly, when one compares it with the socio-economic status (…) then one would see something similar. That it is indeed related.” (1, 73). Yet, intersections of social categories can also change hierarchies of single categories, and education is a marker for an increased perceived status of a racialized person a participant explained: “I would assume, that perhaps a colored man with an academic background is treated better, then a person with a very German name, who has an obvious alcohol abuse issue.” (1,77). Another participant agreed on that: “I see it like this that a dark-skinned person, who has education, is definitely perceived better.” (4, 56). The intersection of race and education can help to increase the perceived status of a person though it does not make them immune for racism. Yet, it might help to decrease prejudice as one non-*white* racialized student remembered from own experience: “Also when I say I study medicine, then I am perceived very differently” (4, 56).

When discussing the intersection of gender and race, the students argued that even a higher economic status does not prevent for example Black women to experience discrimination in childbirth due to their race and gender:

(…) there is an interesting study of the American Heart Association which found out that Afro-American women have a, I think, three to four times higher risk to die of pregnancy complications. And that phenomenon is regardless of their socio-economic background. Also, Serena Williams and Beyoncé are two well-off Black women who suffered through this (3, 16).

Another participant confirmed that birth constitutes a major health risk for Black women: “And that the risk of dying is considerably higher of Black women than of white when giving birth. (…) Also, it is on the one hand the life circumstances and on the other hand of course the care” (4, 67). The student linked the statistically risk of Black women to die during childbirth to intersectional structural racism, such as that their life circumstances are worse than of white women, so that due to racialization they are at higher risk.

One student critically reflected on their experience in the gynaecology division of a hospital: “But definitely women with certain ethnical backgrounds (…) are especially loud when giving birth or especially whiny, more complaining than German or European women and yes that was said frequently” (6, 25). The pain experience of non-white women is systematically downplayed here by medical staff which can lead to serious health complications.

In this way the students summed up the immanent connection between structural racism which has to be seen through an intersectional lens to make the whole scope of it visible. Yet, they also created hierarchies of categories and tried to explain which categories and intersections create greater risks of discrimination than others. This demonstrates a lack of theoretical knowledge about intersectionality and their additive and hierarchal understanding of the intersection of social categories.

## Discussion and conclusion

The participants of the study represent a wide spectrum of knowledge and awareness about structural racism and intersectionality. When some of the students show a rather elaborate knowledge about the concept and effects of structural racism on the individual in medicine, for others it is a new concept they had not considered before but are interested to learn about. In some discussions it is difficult for the participants to decide if a health disparity is based on structural racism or on other factors, for example perceived cultural differences. Yet, as great challenge must be considered to identify and evaluate structural racism in Germany. It is much easier for the participants to discuss this topic in the context of the United States than to transfer it to Germany. The students show difficulties in finding the right terms or they argue that there is no national difference between Germany and the United States regarding structural racism. This problem of addressing structural racism in Germany due to conceptual unclarity is an important finding of this study. The students have problems to transfer the English term race into the German context. On the one hand they try to find substitute terms, such as migration background to describe racialization in the German context. On the other hand, they reason to what extent class and education might be more relevant concepts to describe the appearance of structural racism in Germany.

In context of intersectionality we can also see a spectrum of knowledge and awareness. Some students are familiar with the term and have some theoretical background, when others can explain the content but are not sure about the terminology. For others it is a new concept, but they show interest to learn more about it. When discussing intersectionality, the students identify several social categories that intersect with race, such class, education, religion, gender, and migration which contribute to the structural effects of racism and demonstrate that a one-dimensional view of racism is not sufficient to grasp the full extent of the problem.

Previous studies have demonstrated that the awareness of medical professionals of structural racism and knowledge of intersectionality has an impact on health disparities and the individual health of patients [14, 15, 51, 53]. This impacts the individual patient’s health, because an awareness of the doctor or nurse can prevent structural-based discrimination in context of diagnosis and treatment of illness [54]. Yet, as our study shows medical students in Germany as the next generation doctors lack substantial knowledge about structural and intersectional racism and the impact on health. They report that this knowledge is not subject of their medical studies yet, though the German national catalogue for learning goals (NKLM) aims to include knowledge about diversity and discrimination at least more thoroughly in future medical studies [45, 67, 68]. Yet, so far knowledge and awareness usually come from a personal interest of the students into the subject and is not generated by academic teaching.

Since the impact of structural and intersectional racism on health has been established by research [6, 11], a value can be seen in teaching medical students theoretical concepts and knowledge to raise their awareness and enable them to address and reflect on these issues in their medical practice. Teaching about structural racism can help to shift the focus of medical practitioners from individual behaviour, for example the impact of bad nutrition choices on health, to structural aspects, for example that poverty might impact the access to healthy food. This can a have an effect on diagnosis and treatment of patients, when the medical practitioner is aware of the structural circumstances of the patient which might impact their illness but also can be an obstacle in recovery, so that a treatment can for example be adjusted or additional help can be provided.

The teaching of intersectionality to medical students will increase their knowledge about the diversity of discrimination that cannot always be described in single-axis forms, such as racism or sexism. For example, Muslim women experience very different forms of discrimination in medicine and health care than *white* women but also than Muslim men. Their experiences cannot be described either in sexism nor in racism but at the intersection of both forms of discrimination. Intersectionality provides a tool to make this multidimensional discrimination visible but when medical staff is not aware of the concept and have no knowledge of its use, they will not be able to identify this form of discrimination and acknowledge its effects on their patients’ health.

Lastly, knowledge and awareness about structural and intersectional racism can have a positive impact on the patient-doctor relationship. Patients who have experienced discrimination in medicine and health care before show trust issues in doctors and avoid attending medical help [20, 69, 70]. Medical practitioner would be able to reflect on their own biases towards certain patient groups. By acknowledging the effects of unjust and discriminatory structures on the patient’s health, they would be able to avoid moralized and individualzsed judgements which might have guided their treatment of the patient previously. This can generate new trust of discriminated patients in their doctors and the healthcare system, so that they might seek help earlier and more effectively.

However, there are some obstacles to be aware of to teach structural and intersectional racism in Germany to medical students. In Germany, the academic debate on this topic is very much still in the beginning and is characterized by a great deal of controversy and lack of clarity in concepts. As our data for example shows, notions of structural racism in the context of the United States cannot easily be transferred to the German context due to different terms, such as race which is for historical reasons not used in Germany but inadequately substituted by for example migration background. Also, the groups affected by structural and intersectional racism are very divers in Germany. Especially post-war immigrants, the so-called “guest workers” from Southern Europe and Muslim people, experience racism in general but also in medicine and health care [32, 71]. Intersectionality might function here as theoretical framework that can help to clarify the confusion of concepts due to its multidimensional approach to racism acknowledging the more social categories impacting experiences of racism, such as migration or religion. This diversity must be conceptualized and taught to medical students so that they understand the important nuances and effects on health. Intersectionality is a fruitful concept here to address this diversity of racism but also intersectionality experience strong debates about the appropriate use and inclusion of social categories.

Yet, our study itself included some limitations. As a qualitative-explorative study the sample is small. Though we included students from universities all over Germany, partly only one student from a university is included. Also, the political engagement of the students was mostly on the left and liberal spectrum. A more heterogenous sample would have provided deeper insights than we could gain. Nevertheless, our data clearly shows the ubiquity of the topic in German medicine and health care as well as an interest of medical students in structural racism and intersectionality. We could demonstrate that a lack of teaching in the medical curriculum exists in Germany and that it should be considered to include topics of structural and intersectional racism more in-depth into medical education, because it can help to address health disparities and improve care. Yet, our study cannot provide a ready-made plan for this but rather calls for further research how the topic of structural and intersectional racism can be adequately addressed in medical curricula.

## Data Availability

The datasets used and analysed during the current study are available from the corresponding author on reasonable request.
